# Myocyte [Na^+^]_i_ Dysregulation in Heart Failure and Diabetic Cardiomyopathy

**DOI:** 10.3389/fphys.2018.01303

**Published:** 2018-09-12

**Authors:** Sanda Despa

**Affiliations:** Department of Pharmacology and Nutritional Sciences, University of Kentucky, Lexington, KY, United States

**Keywords:** heart failure, type-2 diabetes, Na^+^-glucose cotransporter, Na^+^/H^+^ exchanger, Na^+^/K^+^-ATPase, Na^+^/Ca^2+^ exchanger

## Abstract

By controlling the function of various sarcolemmal and mitochondrial ion transporters, intracellular Na^+^ concentration ([Na^+^]_i_) regulates Ca^2+^ cycling, electrical activity, the matching of energy supply and demand, and oxidative stress in cardiac myocytes. Thus, maintenance of myocyte Na^+^ homeostasis is vital for preserving the electrical and contractile activity of the heart. [Na^+^]_i_ is set by the balance between the passive Na^+^ entry through numerous pathways and the pumping of Na^+^ out of the cell by the Na^+^/K^+^-ATPase. This equilibrium is perturbed in heart failure, resulting in higher [Na^+^]_i_. More recent studies have revealed that [Na^+^]_i_ is also increased in myocytes from diabetic hearts. Elevated [Na^+^]_i_ causes oxidative stress and augments the sarcoplasmic reticulum Ca^2+^ leak, thus amplifying the risk for arrhythmias and promoting heart dysfunction. This mini-review compares and contrasts the alterations in Na^+^ extrusion and/or Na^+^ uptake that underlie the [Na^+^]_i_ increase in heart failure and diabetes, with a particular emphasis on the emerging role of Na^+^ - glucose cotransporters in the diabetic heart.

## Maintenance of Myocyte Na^+^ Homeostasis is Vital for Preserving Heart Function

All mammalian cells maintain a low intracellular Na^+^ concentration ([Na^+^]_i_) by actively extruding Na^+^ through the Na^+^/K^+^-ATPase (NKA) at the expense of metabolic energy. The energy stored in the electrochemical Na^+^ gradient is then used for the transmembrane transport of other ions (e.g., Ca^2+^ through the Na^+^/Ca^2+^ exchanger, NCX, H^+^ via the Na^+^/H^+^ exchanger, NHE, etc.), uptake of energy substrates (glucose through the family of Na^+^-glucose cotransporters, SGLTs, and aminoacids through Na^+^-aminoacid cotransporter) and, in the case of excitable cells, generation of action potentials (via voltage-gated Na^+^ channels). Changes in [Na^+^]_i_ critically affect the function of these transporters, therefore [Na^+^]_i_ homeostasis is essential for numerous cellular processes.

**FIGURE 1:**
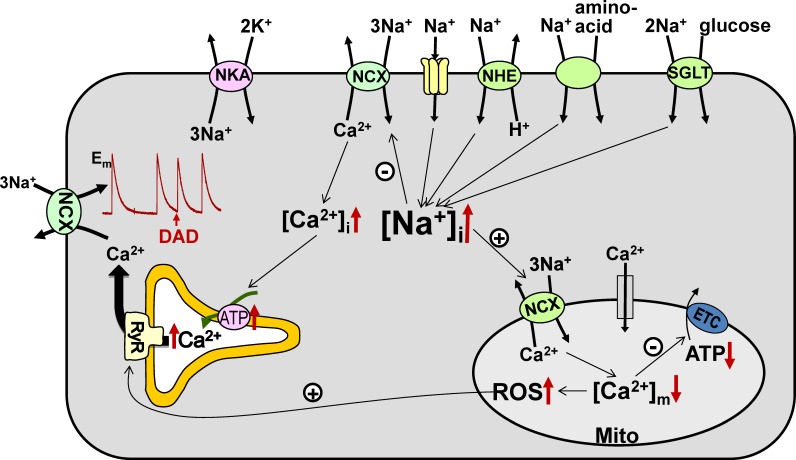
Na^+^ transport pathways and consequences of elevated [Na^+^]_i_ in cardiac myocytes. Several pathways, including the Na^+^/Ca^2+^ exchanger (NCX), Na^+^ channels, Na^+^/H^+^ exchanger (NHE), Na^+^-glucose cotransporter (SGLT), and Na^+^-aminoacid cotransporter, contribute to the entry of Na^+^ into cardiac myocytes, while the Na^+^/K^+^-ATPase (NKA) is the essential route for Na^+^ efflux. Either enhanced Na^+^ influx or impaired Na^+^ extrusion result in higher [Na^+^]_i_. Elevated [Na^+^]_i_ leads to (i) increased cellular and SR Ca^2+^ load, which augments the risk for the occurrence of delayed afterdepolarizations (DADs), and (ii) reduced mitochondrial Ca^2+^ levels, which leads to lower ATP production and causes oxidative stress. ETC – electron transport chain.

In cardiac myocytes, NCX is the main route for Ca^2+^ extrusion from the cells (Bers, 2001), which intimately links Ca^2+^ cycling to [Na^+^]_i_. Even a small (few mM) increase in [Na^+^]_i_ alters Ca^2+^ fluxes through NCX, resulting in higher Ca^2+^ levels in the cytosol and sarcoplasmic reticulum (SR) and consequently larger contractions (**Figure 1**). This mechanism is responsible for the inotropic effect of cardiac glycosides such as digoxin. However, as demonstrated clinically with digoxin, the beneficial effect of enhanced contractility is counteracted by a higher risk for ectopic arrhythmias, as larger SR Ca^2+^ load increases the incidence of spontaneous Ca^2+^ waves and delayed afterdepolarizations (Bers, 2014).

[Na^+^]_i_ also controls the level of Ca^2+^ in the mitochondria ([Ca^2+^]_m_) through the mitochondrial Na^+^/Ca^2+^ exchanger (mitoNCX), which is the main route for mitochondrial Ca^2+^ extrusion in the heart (Griffiths, 2009). MitoNCX is half-maximally activated at [Na^+^]_i_ in the physiological range (5–10 mM) (Boyman et al., 2013). Therefore, mitoNCX is very sensitive to changes in [Na^+^]_i_. An increase in [Na^+^]_i_ accelerates mitochondrial Ca^2+^ efflux and thus reduces [Ca^2+^]_m_ (Cox and Matlib, 1993; Maack et al., 2006). Because [Ca^2+^]_m_ stimulates several dehydrogenases involved in the tricarboxylic acid cycle (McCormack et al., 1990), regeneration of NADH and NADPH from their oxidized forms slows down at lower [Ca^2+^]_m_. Slower restoration of the NADH pool limits the rate of electron transport and thus diminishes mitochondrial ATP production. Notably however, glycolytic ATP also drives cellular processes in the heart, particularly NKA (Glitsch and Tappe, 1993). NADPH is utilized to neutralize the H_2_O_2_ produced by the electron transport chain and lower NADPH levels may result in oxidative stress (Bertero and Maack, 2018). Abnormally low [Na^+^]_i_ is likely to have the opposite effect and cause mitochondrial Ca^2+^ overload, which also has detrimental effects on myocyte function. This notion is supported by the recent finding that elimination of mitochondrial Ca^2+^ efflux through deletion of the gene encoding mitoNCX results in increased generation of superoxide and necrotic cell death leading to heart failure (Luongo et al., 2017).

In summary, [Na^+^]_i_ regulates Ca^2+^ cycling, electrical activity, and oxidative stress in cardiac myocytes. Thus, maintenance of myocyte Na^+^ homeostasis is vital for preserving heart function.

## Myocyte [Na^+^]_i_ is Elevated in Heart Failure and Type-2 Diabetes

[Na^+^]_i_ is in the 4–8 mM range in resting ventricular myocytes from healthy rabbit, guinea-pig, dog and human hearts (Harrison et al., 1992; Yao et al., 1998b; Gray et al., 2001; Despa et al., 2002a; Pieske et al., 2002; Gao et al., 2005) and somewhat higher (10–15 mM) in rat and mouse myocytes (Donoso et al., 1992; Yao et al., 1998a; Despa et al., 2002a, 2005). [Na^+^]_i_ increases in a frequency dependent manner when myocytes are excited electrically. This [Na^+^]_i_ rise is caused by enhanced Na^+^ entry due to the regular opening of the voltage-gated Na^+^ channels and activation of NCX during Ca^2+^ transients. As [Na^+^]_i_ rises, NKA is activated to extrude more Na^+^ and a new steady-state is reached when the higher Na^+^ influx is balanced by an elevated Na^+^ efflux.

The balance between the passive Na^+^ entry and Na^+^ pumping out of the cell is perturbed in both humans and animal models with heart failure (HF), resulting in elevated [Na^+^]_i_. Pieske et al. (2002) found that [Na^+^]_i_ is ∼4 mM higher in myocytes from failing human myocardium compared to non-failing hearts. A comparable [Na^+^]_i_ increase was reported in myocytes from rabbits with heart failure induced by volume and pressure overload (Despa et al., 2002b; Baartscheer et al., 2003) or by rapid pacing (Schillinger et al., 2006). [Na^+^]_i_ is also elevated in myocytes from mice with heart failure caused by conditional, cardiomyocyte-specific deletion of SERCA gene (Louch et al., 2010). These studies found that [Na^+^]_i_ was elevated at all stimulation rates in the 0–3 Hz range. While a few mM rise in [Na^+^]_i_ may seem modest, Despa et al. (2002b) showed that, compared to other HF-induced alterations such as smaller Ca^2+^ transients and longer action potentials, it has the greatest impact on NCX function, and thus on cellular and SR Ca^2+^ load.

Diabetes is a systemic disease that leads to structural, contractile and electrical abnormalities of the heart, even in the absence of coronary artery disease or hypertension (Taegtmeyer et al., 2002; Young et al., 2002, 2009; Guha et al., 2008; Boudina and Abel, 2010). Diabetic cardiomyopathy is characterized by diastolic dysfunction (>50% prevalence) that progresses to systolic dysfunction and heart failure at more advanced diabetic stages (Ingelsson et al., 2005; Kostis and Sanders, 2005; Masoudi and Inzucchi, 2007; Pataky et al., 2011; Chaudhary et al., 2015). Some studies reported alterations in myocyte Na^+^ transport consistent with elevated [Na^+^]_i_ in animal models of both type-1 and type-2 diabetes (see below). However, whether or not [Na^+^]_i_ is altered in diabetic hearts was largely unknown until we recently found higher [Na^+^]_i_ in resting and contracting myocytes from rats with late-onset type-2 diabetes that display a cardiac phenotype that closely resembles the diabetic cardiomyopathy in humans (HIP rats) (Lambert et al., 2015). Interestingly, the [Na^+^]_i_ rise that we measured in diabetic HIP rat hearts is comparable to the increase in [Na^+^]_i_ that occurs in HF.

As discussed above, high [Na^+^]_i_ may amplify the risk for arrhythmias and cause oxidative stress. Indeed, elevated [Na^+^]_i_ was shown to cause oxidation of NAD(P)H and to increase the H_2_O_2_ level in myocytes from guinea-pigs with heart failure induced by aortic constriction (Liu and O’Rourke, 2008). These effects were prevented by pharmacological inhibition of mitoNCX (Liu and O’Rourke, 2008). In a guinea pig model of heart failure and sudden cardiac death (aortic constriction + daily β-adrenergic receptor stimulation), mitoNCX inhibition attenuated cardiac hypertrophic remodeling and prevented cardiac dysfunction and sudden cardiac death, likely through normalizing [Ca^2+^]_m_ (Liu et al., 2014). Therefore, the increase in [Na^+^]_i_ is an active contributor to heart dysfunction in HF. Similar mechanisms are likely also in play in diabetic cardiomyopathy. In support of this assertion, Babsky et al. (2001) found that higher [Na^+^] had a larger negative effect on state 3 respiration, rate of oxidative phosphorylation and ATP production in mitochondria isolated from rats with streptozotocin-induced diabetes compared to control.

## Mechanisms Underlying the [Na^+^]_i_ Rise in Heart Failure and Diabetes

Since [Na^+^]_i_ is at steady-state when the active Na^+^ efflux through the Na^+^/K^+^-ATPase and the total Na^+^ influx through various pathways (**Figure 1**) are at equilibrium, the [Na^+^]_i_ rise in HF and diabetic cardiomyopathy could be caused by both reduced Na^+^ extrusion and enhanced Na^+^ entry.

### Na^+^/K^+^-ATPase Expression and Function in Heart Failure and Diabetes

Numerous studies in human myocardium and animal models reported lower protein expression of various NKA subunits in HF. Schwinger et al. (1999) found reduced expression of NKA α_1_-, α_3_-, and β_1_-subunits in failing human hearts. Protein expression of all three α-subunit isoforms is decreased in hearts from rabbits with pressure and volume overload-induced HF (Bossuyt et al., 2005). In contrast, expression of α_1_ isoform is unchanged while α_2_ is reduced and α_3_ is increased in most rat HF models (Verdonck et al., 2003a). These data suggest that NKA activity might be reduced in HF. However, NKA function depends strongly on regulation by various modulators, including the endogenous inhibitor phospholemman. Indeed, Boguslavskyi et al. (2014) reported hypophosphorylation of phospholemman with no change in NKA expression following aortic constriction in mice, which resulted in a progressive decline in NKA current and elevation of [Na^+^]_i_. Thus, functional measurements in live cells and intact beating hearts are needed in order to compare NKA activity in failing and control hearts. By measuring the rate of [Na^+^]_i_ decline as a function of [Na^+^]_i_ in live myocytes, we found no changes in either the maximal Na^+^ transport rate or the apparent affinity for internal Na^+^ in myocytes from failing rabbit hearts compared to controls (Despa et al., 2002b). Decreased maximal Na^+^ extrusion rate (mainly through NKA-α2 isoform) but unchanged [Na^+^]_i_-affinity were reported in myocytes from rats with HF following myocardial infarction (Semb et al., 1998; Swift et al., 2008) as well as in mice with end-stage HF following genetic deletion of SERCA2 (Louch et al., 2010). In contrast, myocytes from dogs with chronic atrioventricular block and hypertrophy have unaltered maximal NKA current but reduced NKA [Na^+^]_i_-affinity (Verdonck et al., 2003b). Overall, NKA activity is decreased in some but not all HF models investigated, which may contribute to the rise in [Na^+^]_i_.

There are significantly fewer studies of NKA expression and function in diabetic cardiomyopathy. NKA activity is reduced by 21% in the myocardium of rats with streptozocin-induced type-1 diabetes (Kjeldsen et al., 1987). Hansen et al. (2007) found decreased NKA current measured with 10 mM Na^+^ but not with 80 mM Na^+^ in the pipette solutions in myocytes from rabbits with alloxan-induced type-1 diabetes, which indicates a reduction in the affinity of NKA for internal Na^+^ but no change in the maximal NKA activity. Myocytes from type-2 diabetic HIP rats showed no change in NKA-mediated Na^+^ extrusion for [Na^+^]_i_ in the physiological range (0–20 mM) compared to their control littermates (Lambert et al., 2015). In agreement with this result, we also found that NKA-α1 expression is unaltered, while there is ∼50% decrease in NKA-α2 (Lambert et al., 2015). Since NKA-α2 represents less than 25% of the total NKA in rat myocytes (Despa and Bers, 2007; Swift et al., 2007), a ∼50% reduction in its expression has only a minor effect on total NKA function. However, NKA-α2 has a preferential localization in the t-tubules (Despa and Bers, 2007; Swift et al., 2007, 2010; Despa et al., 2012a) and therefore reduced NKA-α2 expression might affect Ca^2+^ cycling and contractility through local, subcellular, effects.

### Na^+^ Influx Pathways in Heart Failure and Diabetes

We (Despa et al., 2002b) and others (Baartscheer et al., 2003) found that higher [Na^+^]_i_ is caused by enhanced Na^+^ influx rather than reduced NKA activity in hearts from rabbits with pressure and volume-overload induced HF. This is also the case in myocytes from type-2 diabetic HIP rats, where Na^+^ influx is increased by ∼40% compared to myocytes from control rats (Lambert et al., 2015). While not directly measuring the rate of Na^+^ entry, several other studies reported upregulation of membrane transporters that facilitate Na^+^ import in HF and diabetic cardiomyopathy (see below), supporting an essential role for Na^+^ influx in the [Na^+^]_i_ rise that occurs in these pathological conditions.

#### Na^+^ Current in Heart Failure and Diabetes

We found that the excess Na^+^ influx in myocytes from rabbits with HF is TTX-sensitive, which suggests that it is carried by Na^+^ channels (Despa et al., 2002b). There are numerous reports of increased late Na^+^ current in HF (Maltsev et al., 1998; Valdivia et al., 2005; Mishra et al., 2015; Hegyi et al., 2018), a slowly inactivating current that may be carried by both cardiac and neuronal Na^+^ channels present in myocytes. While the amplitude of late Na^+^ current is small (∼0.1–0.5% of the amplitude of the peak Na^+^ current), the current is long lasting (hundreds of milliseconds) and thus may contribute to myocyte Na^+^ homeostasis. However, the role of late Na^+^ current in elevating Na^+^ influx and [Na^+^]_i_ in HF is controversial. On one hand, ranolazine, a late Na^+^ current inhibitor, reduced [Na^+^]_i_, and diastolic Ca^2+^ overload in failing human hearts (Sossalla et al., 2008). Moreover, the CaMKII-dependent increase in late Na^+^ current produced by exogenous reactive oxygen species was associated with a TTX and ranolazine-dependent rise in [Na^+^]_i_ (Wagner et al., 2011). On the other hand, computational modeling predicts that higher late Na^+^ current measured in myocytes from failing hearts generates only a modest increase in [Na^+^]_i_, smaller than measured experimentally (Wagner et al., 2011; Cardona et al., 2016). Alternatively, HF may also enhance a background Na^+^ channel conductance that is responsible for the higher rate of Na^+^ entry.

Independent of a potential effect on [Na^+^]_i_, increased late Na^+^ current contributes to the prolongation of the action potential in HF, which may result in early afterdepolarizations. Moreover, via NCX, longer action potentials favor Ca^2+^ loading of the myocyte, which increases the propensity for delayed afterdepolarizations. Indeed, ranolazine significantly abbreviated the action potential and prevented the occurrence of delayed afterdepolarizations in myocytes from mice with HF induced by aortic constriction (Toischer et al., 2013). Ranolazine prevented ventricular fibrillation in rabbits with pacing-induced HF (Frommeyer et al., 2012) Thus, while the contribution of increased late Na^+^ current to the rise in [Na^+^]_i_ is not fully elucidated, late Na^+^ current inhibition has proven beneficial effects in HF.

#### Na^+^/Ca^2+^ Exchanger in Heart Failure and Diabetes

NCX, which exchanges three Na^+^ ions for one Ca^2+^, is the main route for Ca^2+^ extrusion (Bers, 2001) and the most prominent contributor to Na^+^ influx (Despa et al., 2002a) in cardiac myocytes. Cardiac NCX expression is generally increased in both animal models of HF (O’Rourke et al., 1999; Pogwizd et al., 1999; Louch et al., 2010) and failing human hearts (Hasenfuss et al., 1999). However, higher NCX expression does not necessarily translate into higher rate of NCX-mediated Na^+^ entry. This is because the higher [Na^+^]_i_ and smaller Ca^2+^ transients typically seen in HF shift the balance of fluxes through NCX to disfavor the Ca^2+^ out/Na^+^ in mode of function and may even cause the reversal of the exchanger during an action potential. In agreement with this reasoning, we found no change in the NCX-mediated Na^+^ influx in failing rabbit myocytes (Despa et al., 2002b) despite a 100% increase in NCX expression (Pogwizd et al., 1999).

In contrast to HF, NCX expression and function is decreased in rats with streptozotocin-induced type-1 diabetes (Chattou et al., 1999; Hattori et al., 2000) and in type-2 diabetic HIP rats (Despa et al., 2012b). It is thus unlikely that NCX contributes to the enhanced myocyte Na^+^ entry in diabetes.

#### Na^+^/H^+^ Exchanger in Heart Failure and Diabetes

NHE is markedly upregulated in HF (Yokoyama et al., 2000; Leineweber et al., 2007; Fliegel, 2009; Packer, 2017) and its inhibition improved heart function in various animal models of HF (Kusumoto et al., 2001; Engelhardt et al., 2002; Aker et al., 2004; Kilić et al., 2014). Baartscheer et al. (Baartscheer et al., 2003) reported that increased Na^+^/H^+^-exchange activity is responsible for elevated [Na^+^]_i_ in myocytes from failing rabbit hearts. Moreover, chronic treatment with cariporide, an NHE inhibitor, prevented the onset of HF in rabbits with pressure and volume overload (Baartscheer et al., 2005). The activity of myocardial Na^+^/H^+^ exchanger (NHE) is enhanced and contributes to left ventricular hypertrophy in the Goto-Kakizaki rat model of T2D (Darmellah et al., 2007). Increased NHE activity leads to higher [Na^+^]_i_ gain during ischemia-reperfusion in hearts from T2D db/db mice (Anzawa et al., 2006). Moreover, reducing [Na^+^]_i_ gain during ischemia-reperfusion by NHE inhibition was associated with a lower incidence of ventricular tachycardia and fibrillation in db/db hearts (Anzawa et al., 2006). These data point to an important contribution of NHE to the excess cardiac Na^+^ influx in HF and diabetic cardiomyopathy.

## Na^+^-Glucose Cotransporter and [Na^+^]_i_ Dysregulation in Diabetic Hearts

One transporter known to be present in the heart but rarely discussed in the context of myocyte Na^+^ homeostasis is the Na^+^-glucose cotransporter (SGLT), which couples Na^+^ transport to glucose uptake and thus to energy substrate metabolism. The major SGLT isoforms, SGLT1, and SGLT2, have distinct tissue distribution and systemic role. SGLT1 is found predominantly in epithelial cells from the intestine, where it participates in dietary glucose absorption, whereas SGLT2 is the major isoform expressed in renal epithelial cells and is essential for glucose reabsorption from the forming urine. Highly specific SGLT2 inhibitors are the latest class of blood glucose lowering drugs. Recently, SGLT2 inhibitors were demonstrated to have beneficial cardiac effects in patients with type-2 diabetes and HF (Zinman et al., 2015). However, reports from several labs (Nishimura and Naito, 2005; Wright and Loo, 2011; Van Steenbergen et al., 2017) indicate that SGLT2 is not expressed in the heart. This suggests that the cardioprotection conferred by SGLT2 inhibitors is mediated by interaction with a different cardiac target and/or by effects in extracardiac tissues. Intriguingly, two recent studies (Baartscheer et al., 2017; Uthman et al., 2018) found that SGLT2 inhibitors (empagliflozin, dapagliglozin, and canagliflozin) block the Na^+^/H^+^ exchanger, possibly by binding to the Na^+^-binding site of NHE, and thus lower myocyte [Na^+^]_i_ in multiple species. Furthermore, empagliflozin and canagliflozin also induced vasodilation (Uthman et al., 2018). While these measurements were performed in healthy hearts/myocytes, the effects uncovered are likely to play a part in the improvement of heart function by SGLT2 inhibitors that was observed clinically in patients with HF and type-2 diabetes.

In contrast to SGLT2, SGLT1 is highly expressed in the heart (Zhou et al., 2003; Banerjee et al., 2009, 2010; Wright and Loo, 2011). Recently, SGLT1 upregulation was causally linked to PRKAG2 cardiomyopathy that is caused by mutations in the gene encoding the γ2 subunit of AMP-activated protein kinase (Banerjee et al., 2010) and cardiac-specific SGLT1 deletion attenuated the cardiomyopathy (Ramratnam et al., 2014). Moreover, cardiac-specific overexpression of SGLT1 in mice causes hypertrophy and left-ventricular dysfunction (Ramratnam et al., 2014). Thus, there is increasing evidence that enhanced SGLT1 activity harms the heart.

Banerjee et al. (2009) reported that the mRNA level of SGLT1 is increased in hearts from humans and mice with type-2 diabetes and ischemic cardiomyopathy. In agreement with this result, we found higher SGLT1 protein expression in failing hearts from T2D patients compared to failing hearts from lean, non-diabetic individuals and in hearts from type-2 diabetic HIP rats vs. control rats (Lambert et al., 2015). Obesity, in the absence of type-2 diabetes, was also associated with elevated levels of cardiac SGLT1 in human hearts (Lambert et al., 2015). Moreover, the presence of HF alone resulted in higher cardiac SGLT1 protein expression in both lean and obese individuals (Lambert et al., 2015). Consistent with augmented SGLT1 expression, we found that the SGLT-mediated glucose uptake is significantly increased in hearts from diabetic vs. control rats (Lambert et al., 2015). Moreover, SGLT inhibition (both pharmacological and through omission of glucose from external solution) greatly reduced the rate of Na^+^ entry in myocytes from diabetic rats while it had no significant effect in myocytes from control rats. SGLT-mediated Na^+^ influx was ≈7 times higher in diabetic vs. control hearts (Lambert et al., 2015). Together, these results indicate that SGLT1 is upregulated in diabetic rat hearts and its activation is largely responsible for the excess Na^+^ influx that accounts for the increase in [Na^+^]_i_.

Other members of the Na^+^-glucose cotransporter family may also play a role in the development of diabetic cardiomyopathy. Van Steenbergen et al. (2017) found that hyperglycemia stimulates the production of reactive oxygen species in the heart through activation of NADPH oxidase and this mechanism is prevented in myocytes from mice with genetic deletion of the sodium-myoinositol cotransporter-1 (SMIT1). This finding suggests that SMIT1 is activated by high glucose, which may also contribute to the [Na^+^]_i_ rise in diabetic hearts.

In summary, [Na^+^]_i_ is augmented in both HF and diabetic cardiomyopathy and higher [Na^+^]_i_ further exacerbates heart dysfunction. An enhancement of Na^+^ influx is at least partially responsible for the [Na^+^]_i_ rise in both conditions. However, the Na^+^ entry pathways that mediate the excess Na^+^ entry may be distinct in the two pathologies, with Na^+^ channels and NHE being activated in HF and SGLT isoforms and NHE having the main contribution in diabetic cardiomyopathy.

## Author Contributions

SD reviewed the literature in the field, and wrote and edited the manuscript.

## Conflict of Interest Statement

The author declares that the research was conducted in the absence of any commercial or financial relationships that could be construed as a potential conflict of interest.
